# Post-Transcriptional Regulatory Network of Non-Coding RNAs in Yaks: Molecular Mechanisms of Hypoxia Adaptation and Productive Traits

**DOI:** 10.3390/ani16131981

**Published:** 2026-06-26

**Authors:** Huanyu Guan, Wen Hu, Shuo Zhu, Du’an Chen, Zhuoying Zhao, Hui Wang, Jiabo Wang, Binglin Yue, Jincheng Zhong, Jikun Wang

**Affiliations:** Key Laboratory of Qinghai-Tibetan Plateau Animal Genetic Resource Reservation and Utilization, Sichuan Province and Ministry of Education, Southwest Minzu University, Chengdu 610225, China; 17208276933@163.com (H.G.); 18398573987@163.com (W.H.); 16687104023@163.com (S.Z.); 13982634140@163.com (D.C.); zhao19252026@163.com (Z.Z.); wanghui892321@swun.edu.cn (H.W.); wangjiaboyifeng@163.com (J.W.); yuebinglin123@163.com (B.Y.); zhongjincheng518@126.com (J.Z.)

**Keywords:** yak, non-coding RNA, high-altitude adaptation, multi-omics

## Abstract

Yaks have long inhabited the Qinghai-Tibetan Plateau. This region features low-oxygen, frigid temperatures and pronounced seasonal variation in nutrient availability. They have evolved adaptive phenotypes centered on energy metabolism reprogramming, tissue structure remodeling, and stress homeostasis maintenance. In recent years, non-coding RNAs (ncRNAs) have been confirmed as an important component of the yak’s post-transcriptional regulatory network. They play a key bridging role between environmental stress perception and phenotypic output through mechanisms such as influencing RNA splicing, stability, translation activity, and constructing competitive endogenous RNA (ceRNA) networks. This article systematically reviews the biogenesis pathways and core regulatory patterns of circular RNAs (circRNAs), microRNAs (miRNAs), and long non-coding RNAs (lncRNAs). It focuses on summarizing the expression profile characteristics and dynamic spatiotemporal changes of these three types of ncRNAs in physiological contexts such as muscle and fat deposition, mammary gland lactation, testicular development, and hypoxia response in the heart, lungs, and vascular system of yaks. Current research evidence indicates that the regulatory network of yaks ncRNAs shows significant convergence on multiple key signaling pathways, mainly concentrating on lipid metabolism (PPAR/AMPK), nutrition and growth signals (PI3K-Akt/MAPK/mTOR), extracellular matrix remodeling (ECM-receptor interaction, Wnt/TGF-β), and cell stress fate determination (apoptosis, oxidative stress/ferroptosis) modules. Among them, some core circRNA and lncRNA-miRNA-mRNA regulatory axes have been functionally validated in vitro. Despite the phased progress, current research on ncRNA in yaks still faces bottlenecks: the multi-omics molecular atlases (encompassing genomics, transcriptomics, proteomics, and metabolomics) of key high-altitude adaptive organs remain incomplete, analysis processes lack sufficient standardization, and most studies stay at the association network level with limited causal mechanism validation. To address these limitations, future research should focus on building a standardized evidence chain, integrating multi-omics and single-cell/spatial transcriptome technologies, and conducting mechanism verification for traits in independent populations, thereby providing a solid theoretical basis for understanding the extreme environmental adaptation mechanisms of yaks and molecular breeding improvement.

## Simple Summary

Yaks are uniquely adapted to survive in the harsh environment of the Qinghai-Tibetan Plateau, where oxygen is scarce, temperatures are freezing, and food availability changes drastically with seasons. Understanding how these animals cope with such extreme conditions is crucial for both conservation and agriculture. This review explores the hidden biological mechanisms behind the yak’s remarkable resilience, focusing on a special type of genetic material called non-coding RNAs (ncRNAs). Unlike regular genes, these molecules act like master switches, controlling how the yak’s body responds to stress and builds important tissues. We examined how these genetic switches influence the animal’s ability to store fat and muscle, produce milk, develop reproductive organs, and withstand low oxygen levels in the heart and lungs. Our findings reveal that these ncRNAs regulate key biological pathways related to energy usage, cell protection, and tissue structure. By mapping out these genetic controls, this study provides valuable insights that can help scientists breed healthier yaks and better understand how large animals adapt to extreme climates.

## 1. Introduction

Non-coding RNA (ncRNA), as a core molecule in post-transcriptional regulation of genes, has become a key to understanding the complexity of gene expression. While transcriptional regulation has traditionally received the most attention, an increasing number of studies have shown that the post-transcriptional regulation process (involving RNA processing, modification, localization, translation and degradation) is also a crucial stage in determining the final expression of genes [1,2]. During this process, ncRNA precisely regulates the spatiotemporal dynamics of gene expression through the synergistic action with RNA-binding proteins, mRNA structural elements and RNA modification enzymes [3,4]. Moreover, a large amount of evidence indicates that abnormal expression of ncRNA is closely related to the occurrence and development of various diseases, such as mental disorders, tumors, cardiovascular and metabolic disorders [5,6].

Circular RNA (circRNA) is a special type of non-coding RNA formed through the reverse splicing of exons or introns. Its main feature is the lack of free 5′ and 3′ ends, presenting a covalently closed loop structure [7]. circRNA plays a significant role in gene expression and functional regulation. Given that ncRNAs have been widely confirmed to be involved in the disease pathogenesis, physiological regulation, and environmental response of other mammals [8,9], conducting research on ncRNA in yaks has a solid theoretical foundation. Compared with linear RNA, circRNA, which is widely present in eukaryotic cells, is less prone to degradation by ribonucleases, and has higher stability, sequence conservation, and tissue specificity. It is a highly potential molecular biomarker and an emerging hotspot in the current RNA research field [10,11,12,13].

MicroRNAs (miRNAs) act as negative regulatory factors in post-transcriptional gene silencing and play a significant role in the adaptive responses of mammals [14,15]. Moreover, the discovery of miRNA’s nuclear functions has further broadened our understanding of their subcellular localization and mechanism of action [14]. Studies have shown that miRNAs are widely involved in the stress response and maintenance of physiological homeostasis in animals. Considering the strong hypoxia tolerance and immune regulation ability of yaks in the hypoxic plateau environment, it is speculated that these molecules also play a key regulatory role in high-altitude environmental stress, which is worthy of in-depth exploration.

Long non-coding RNAs (lncRNAs) play significant roles in regulating gene transcription, chromatin remodeling, and RNA processing and modification [16,17]. Studies have confirmed that lncRNAs can form competitive regulatory axes by binding to miRNAs, thereby influencing cell proliferation and anti-apoptotic capacity [16,17]. Given that yaks are constantly exposed to significant seasonal climate and nutritional fluctuations, it indicates that there exists a complex environmental response and metabolic regulatory network mediated by lncRNAs within their bodies.

As a unique domestic animal of the Qinghai-Xizang Plateau, yaks have long been exposed to extreme environmental stresses such as hypoxia and cold, which impose extremely high demands on their energy metabolism and tissue functions. During their long-term evolution, yaks have developed multi-level hypoxia adaptation mechanisms, among which lipid metabolism reprogramming plays a key role. In recent years, multi-omics studies such as metabolomics, transcriptomics, and microbiomics have significantly deepened our understanding of the hypoxia adaptation mechanisms of yaks. Particularly, research in the field of ncRNA has provided a new perspective for exploring their efficient energy utilization mechanisms [18,19,20], and also contributed important references for the studies of other plateau animals in aspects such as oxygen sensing, lipid metabolism regulation, and microecological interaction [21,22]. In summary, this article will systematically review the research progress of non-coding RNAs such as circRNA, miRNA, and lncRNA in recent years, and focus on discussing their molecular regulatory mechanisms in the high-altitude adaptation and production trait development of yaks, aiming to provide a theoretical basis and new ideas for understanding the adaptive evolution of yaks in extreme ecological environments and promoting the improvement of plateau characteristic animal husbandry.

## 2. Methods

### 2.1. Literature Search Strategy

This review was conducted following the standard guidelines for narrative reviews. A comprehensive literature search was performed to identify relevant studies on non-coding RNAs (ncRNAs) in yaks (*Bos grunniens*). The primary databases used for the search included PubMed (National Center for Biotechnology Information, Bethesda, MD, USA), Web of Science (Clarivate, London, UK), Scopus (Elsevier, Amsterdam, The Netherlands), and China National Knowledge Infrastructure (CNKI) (Tsinghua Tongfang, Beijing, China). Figures were created using BioRender (https://www.biorender.com/, BioRender Inc., Toronto, ON, Canada). This review did not use any professional software, equipment, or chemical reagents other than those mentioned above during the literature search and data integration process.

The following keywords and their combinations were used: “Yak” OR “*Bos grunniens*”, AND “Non-coding RNA” OR “ncRNA”, AND “High-altitude adaptation” OR “Hypoxia”, AND “ceRNA network”, “circRNA”, “miRNA”, “lncRNA”, “lipid metabolism”, “muscle development”, and “ferroptosis”. The search covered all studies published up to May 2026, with a primary focus on articles published in the last five years to ensure the inclusion of the most recent high-throughput sequencing data and mechanistic validations.

### 2.2. Study Selection and Data Extraction

The initial screening was based on titles and abstracts to exclude irrelevant studies, such as those focusing solely on cattle or other bovine species without direct comparative data on yaks. Full-text articles were retrieved for detailed evaluation if they met the following inclusion criteria: (1) studies involving yak tissues or cells (e.g., heart, lung, muscle, adipose tissue, testis); (2) research providing experimental validation (e.g., qRT-PCR, dual-luciferase assays, RIP) of ncRNA function; (3) omics studies (transcriptomics, metabolomics) related to hypoxia response or economic traits.

Data regarding the experimental models, sequencing platforms (Illumina, Nanopore), key differentially expressed ncRNAs, targeted signaling pathways (e.g., PI3K-Akt, PPAR, HIF-1), and phenotypic outcomes were extracted and synthesized. Special attention was paid to studies that established causal links between ncRNA regulation and yak-specific adaptive phenotypes.

## 3. Overview of the Generation and Mechanisms of Non-Coding RNAs

Non-coding RNAs (ncRNAs) encompass a variety of transcripts with diverse biogenesis pathways and regulatory modes. To systematically interpret the molecular regulatory network of ncRNAs in yaks, this section first elaborates on the generation mechanisms and core functional modes of circRNAs, miRNAs, and lncRNAs to establish a unified theoretical framework. Meanwhile, this article systematically reviews the mainstream research strategies for ncRNA identification and the analysis of their targeted interaction networks (including high-throughput sequencing, bioinformatics prediction, and multi-level experimental validation), which is of significant methodological significance for clarifying the relationship between phenotypic associations and causal mechanisms in related literature.

### 3.1. Circular RNAs: Functionally Specific Non-Coding RNAs Formed by Back-Splicing

Circular RNAs (circRNAs) are a unique class of endogenous non-coding RNAs characterized by a covalently closed loop structure, lacking 5′ caps and 3′ poly-A tails [23]. They are widely expressed in eukaryotes and play critical roles in gene regulation, often acting as microRNA sponges, protein scaffolds, or transcriptional regulators. Although the functional characterization of circRNAs in yaks remains limited, extensive studies in model organisms and other livestock have revealed their pivotal roles in physiological processes such as hypoxia adaptation and muscle development. Therefore, integrating these cross-species insights is essential for understanding the potential regulatory networks governing yak-specific traits. Current research on the mechanism of back-splicing mainly focuses on the “intron pairing model” and the “RNA-binding protein (RBP) driven model”.

The intron pairing model proposes that reverse complementary sequences in flanking introns form stable secondary structures via base pairing, which juxtaposes splicing sites and facilitates circRNA circularization [24]. Relevant vector experiments using actin intron sequences have verified that intron length is significantly positively correlated with circularization efficiency [25].

In contrast to the intron-pairing mechanism, the RBP-driven model highlights that specific RBPs aggregate around splicing sites to dynamically modulate back-splicing events [26]. Systematic studies have shown that RBPs can function both by regulating splicing factors and by directly binding to back-splicing sites [27]. Notably, RBPs perform dual functions throughout the life cycle of circRNAs: they can promote circularization and also bind to mature circRNAs to regulate their stability and downstream functions. Such RBP regulation exhibits obvious cell and tissue specificity. For instance, the SFPQ protein specifically regulates the expression of circRNAs from synaptic-related genes during neural differentiation [28], while Bcas2 is involved in the alternative splicing regulation of antibody class switching [29]. In recent years, deep learning algorithms have been widely applied to predict RBP binding sites on circRNAs, providing a powerful tool for exploring the regulatory networks of unknown RBPs [30].

Regarding their cellular localization and functions, exon-derived circRNAs are primarily enriched in the cytoplasm, where they typically act as miRNA “sponges” (competing endogenous RNAs, or ceRNAs) or regulate signaling pathways by interacting with specific proteins [31,32]. For example, the circRNA derived from the Foxo3 exon can delay the cell cycle by recruiting p21 and CDK2 to form a ternary complex [31]. Additionally, the circRNA from the O-GlcNAc transferase (OGT) exon can stabilize the FOXC1 protein and interfere with ferroptosis in neuroblastoma [32]. In contrast, intron-derived circRNAs mostly remain in the nucleus and participate in transcriptional regulation. For instance, circTTN can recruit PURB protein to inhibit the transcription and myogenic differentiation of the host gene [33], while circGNG7 can block the phosphorylation of HSP27 in head and neck squamous cell carcinoma [34]. The application of high-purity circRNA isolation technology (RPAD) has further revealed the existence of a large number of intron-derived circRNAs, highlighting their extensive biological significance in nuclear regulation [35].

### 3.2. MicroRNAs: Biogenesis and Post-Transcriptional Gene Silencing

The biogenesis of microRNAs (miRNAs) is highly dependent on two conserved endonucleases, Drosha and Dicer [36]. In the canonical pathway, the primary transcript (pri-miRNA) is first cleaved in the nucleus by the Drosha-DGCR8 complex to form the precursor miRNA (pre-miRNA), which is then transported to the cytoplasm by EXPORTIN5 and further cleaved by Dicer to generate the mature miRNA duplex [37,38]. The non-canonical pathway includes the mirtron pathway, which originates from introns and bypasses Drosha cleavage, directly relying on the RNA splicing machinery to generate pre-miRNA [39]. Additionally, some RBPs also participate in regulating the processing efficiency and sequence specificity of miRNAs, constituting an important molecular basis for the non-canonical pathway [37]. Cross-species studies have shown that there is some overlap in the generation pathways of miRNAs and other small RNAs (such as piRNAs and siRNAs) [40]. For example, in C. elegans, the RNA interference pathway involving Dicer and RDE-1 has confirmed the interaction and synergy between miRNAs and siRNAs in processing, assembly, and target silencing [41].

Mature miRNAs are critical regulators of physiological homeostasis and developmental regulation in the body. For instance, in reproductive development, the absence of Drosha or Dicer leads to a comprehensive suppression of miRNAs, which severely hinders the formation of primordial germ cells and sperm [40]. Mature single-stranded miRNAs are loaded onto the Argonaute (AGO) protein to form the miRNA-induced silencing complex (miRISC), which recognizes the 3′UTR region of the target mRNA through base complementarity, thereby mediating translational inhibition or mRNA degradation [42,43]. AGO1, as an evolutionarily conserved core effector protein in both animals and plants, can precisely distinguish miRNAs from siRNAs and regulate their abundance, directly determining the efficiency of gene silencing [44]. In animal models, the silencing of AGO1 in prostate cancer cells has profoundly revealed the intrinsic connection between miRNA dysregulation and tumorigenesis [45]; meanwhile, mammalian miRNAs typically exert translational inhibition through incomplete complementarity [38,46]. Moreover, the loading efficiency of miRISC is also regulated by other factors in the post-transcriptional network, such as the Integrator complex [43]. In terms of miRNA metabolic turnover, the target-directed miRNA degradation (TDMD) mechanism mediated by ZSWIM8 endows cells with the ability to rapidly and dynamically respond to environmental fluctuations [47]. The application of Ago cross-linking immunoprecipitation (CLIP) technology in the fruit fly model has provided direct experimental evidence for the precise localization of functional miRNA::mRNA interaction sites at the whole-genome level [46].

In recent years, the continuous expansion of miRNA research methods has provided solid technical support for the in-depth analysis of their mechanisms. High-throughput small RNA sequencing (sRNA-seq) is the mainstream method for identifying miRNA types and abundances and is widely used in cross-species comparisons and disease evolution analysis [46]. Target prediction often relies on seed sequence complementarity and evolutionary conservation algorithms, and the accuracy can be significantly improved by integrating with CLIP and other omics experiments [46]. At the level of targeted validation, the dual-luciferase reporter system, with its high sensitivity and specificity, remains a classic method for confirming the direct interaction between miRNA and target mRNA [39]; meanwhile, the development of specific reporter systems has also provided new tools for exploring the non-classical maturation pathways of intron-derived miRNAs [39]. Additionally, miRNA research using exosomes as carriers has revealed their potential in intercellular communication and tissue regeneration among cells and tissues [48]. At the system level, the joint analysis of miRNA with circRNA and lncRNA to explore the ceRNA network mechanism they construct during physiological stress or pathological occurrence has become an important paradigm in current non-coding RNA research [49].

### 3.3. Long Non-Coding RNAs: Biogenesis Mechanisms and Multi-Level Regulatory Patterns

Long non-coding RNAs (lncRNAs) are RNA molecules with a transcript length exceeding 200 nucleotides and lacking an effective open reading frame. In terms of biogenesis, the vast majority of lncRNAs are highly similar to protein-coding mRNAs, mainly transcribed by RNA polymerase II (Pol II), and undergo processing steps such as 5′ capping, 3′ polyadenylation, and alternative splicing within the nucleus. However, compared to mRNAs, lncRNAs typically have lower transcriptional efficiency and abundance, and poorer sequence conservation, but exhibit extremely high tissue and spatiotemporal expression specificity. The diversity of their biogenesis pathways and splicing patterns endows lncRNAs with complex secondary structures and flexible subcellular localization, laying a structural foundation for their multi-dimensional gene regulatory functions.

In the gene expression network, lncRNAs mainly exert regulatory effects through cis and trans modes. Cis-regulating lncRNAs are usually located near their transcription start sites and regulate the expression of neighboring genes by influencing local chromatin conformation or interfering with the assembly of transcriptional complexes. For instance, in acute myeloid leukemia, the extensive binding of lncRNAs to chromatin is mostly in a cis mode, significantly affecting the transcriptional activity of key target genes [50]; in genomic imprinting, specific lncRNAs cooperatively regulate the chromatin structure of alleles, ensuring parent-of-origin-specific expression [51]. In the viral immune response, lncRNAs can regulate local immune genes (such as IFI6, TBK1) through cis and distant signaling molecules (such as IRF3, STING) through trans, constructing a fine feedback network that can both amplify antiviral effects and prevent excessive immune responses [52,53]. Additionally, antisense lncRNAs can also have both cis and trans dual functions, playing a key role in regulating embryonic pluripotency and cell differentiation [54].

At the epigenetic level, lncRNAs can act as guide molecules or molecular scaffolds, specifically recruiting epigenetic modification enzyme complexes to specific loci, thereby precisely regulating histone modifications and DNA methylation states [55]. For example, during tumorigenesis, specific lncRNAs can mediate chromatin remodeling and m6A methylation modifications in enhancer regions, profoundly influencing the transcriptional activity of oncogenes [56]. Moreover, this regulation mechanism based on chromatin conformation changes is also common in genomic imprinting domains, where lncRNAs precisely control gene expression by altering the higher-order structure of allelic chromatin [51].

Transcriptional interference is another important regulatory dimension of lncRNAs, where the transcription process of lncRNAs itself can directly inhibit or block the transcription of target genes. In mammalian systems, the transcriptional activity of antisense lncRNAs can effectively interfere with the expression of neighboring genes on the sense strand through mechanisms such as spatial hindrance [54]. This mechanism has significant biological implications in various pathological processes. For instance, differential expression analysis of hippocampal tissues in patients with Alzheimer’s disease suggests that specific lncRNAs are highly likely to be involved in the process of neurodegenerative diseases through transcriptional interference mechanisms [57,58]. At the post-transcriptional level, lncRNAs located in the cytoplasm widely act as ceRNAs. They exert a “sponge” effect by competitively binding and sequestering specific miRNAs, thereby relieving the translational inhibition or degradation of downstream target mRNAs by miRNAs. For example, in Alzheimer’s disease models, the complex ceRNA network composed of lncRNA–miRNA–mRNA has been confirmed to be a key regulatory center for intervening in the homeostasis of the nervous system [57]. Similarly, in the study of atherosclerosis, lncRNAs such as ANRIL finely regulate vascular inflammatory responses and the proliferation of smooth muscle cells through the ceRNA mechanism [59]. This ceRNA interaction model provides an important molecular theoretical basis for understanding the gene network regulation in mammals under complex physiological stress and environmental adaptation.

## 4. Yaks circRNAs: Current Progress and Mechanistic Insights

This section will systematically review the expression profiles and spatiotemporal dynamics of circRNAs in various tissues and research models of yaks, with a focus on the ceRNA regulatory axes related to adipogenesis and skeletal muscle development. It will deeply analyze the role of circRNAs in shaping key phenotypes such as muscle development and lipid deposition, and summarize their potential functions in hypoxia response pathways. Meanwhile, by integrating multi-omics analysis, it will explore the latest progress and challenges in this field. At the methodological level, current yak circRNA research generally adopts the mature analytical framework established in model animals, which combines circRNA identification based on high-throughput RNA sequencing, regulatory network inference, and targeted molecular validation techniques. This approach not only ensures the horizontal comparability among different studies but also further highlights the necessity of establishing standardized mechanism validation strategies in the future.

### 4.1. Expression Profiles and Spatiotemporal Dynamics of circRNAs in Yaks

Circular RNAs (circRNAs), with their covalently closed loop structure conferring high stability and significant tissue- and time-specificity, have emerged as important post-transcriptional regulatory candidates for understanding the formation of complex traits and adaptation to extreme environments in yaks. Current studies mainly rely on whole transcriptome sequencing technology to initially construct circRNA expression profile resources in multiple tissues and physiological contexts (Table 1), laying a solid data foundation for subsequent core molecular screening and functional mechanism analysis.

A review of existing literature reveals that yak circRNA profiling studies have mainly focused on skeletal muscle, adipose tissue, mammary glands, and the male reproductive system, and have shown high condition dependence and stage dynamics (Table 1).

In studies related to nutritional environment and meat quality, yak longissimus dorsi muscles under different feeding systems exhibit significant circRNA differential expression profiles, with these differentially expressed molecules significantly enriched in key biological processes such as insulin signaling, mTOR pathway, and lipid metabolism. Additionally, the combined analysis of whole transcriptomes of paired muscle and adipose tissues also indicates that tissue-specific circRNA differential expression is closely related to lipid storage, myogenesis, PI3K-Akt signaling network, and extracellular matrix (ECM) remodeling. Research on intramuscular fat (IMF) deposition further confirms that the abnormal expression of specific circRNAs is deeply associated with lipid metabolism and mitochondrial function networks; however, some studies have not clearly reported the total number of identified circRNAs in their results (Table 1), which to some extent limits cross-study quantitative comparisons.

During the developmental and physiological cycle progression, yak circRNAs exhibit particularly prominent temporal dynamic characteristics. For instance, time-series studies of preadipocyte differentiation show that circRNA expression levels undergo significant stage-specific changes during the differentiation process and are highly enriched in adipogenesis, Wnt signaling, ECM–receptor interaction, and TGF-β pathways, suggesting that circRNAs are deeply involved in the proliferation–differentiation fate transition and microenvironment remodeling associated with adipogenesis. In mammary gland tissue, there are a large number of differentially expressed circRNAs between the lactation and dry periods, mainly enriched in prolactin signaling, tight junctions, and immune regulation modules, suggesting that they may play a key role in maintaining lactation function and tissue remodeling during the lactation cycle. Moreover, two sets of multi-stage transcriptome data for testis development (from fetal to adult, and from 3 to 24 months of age) both confirm that circRNAs are highly dynamically regulated during yak sexual maturation and spermatogenesis, and are widely involved in core events such as germ cell development, steroid hormone synthesis, meiosis, and post-transcriptional processing. It is particularly worth noting that the differential expression profiles of circRNAs in the epididymis tissues of yaks and yak–donkey hybrids provide valuable post-transcriptional regulatory clues for understanding the mechanism of male sterility caused by distant hybridization (Table 1). Although the circRNA atlas research of yaks has covered multiple key tissues, there are still three bottlenecks that need to be urgently broken through in the current field: Firstly, the tissue sampling is heavily biased towards economically important traits and reproductive-related organs, while for core organs such as the heart and lungs, which play a decisive role in adaptation to high-altitude hypoxia, a systematic circRNA expression catalogue has not yet been constructed. Secondly, there is heterogeneity in sample experimental grouping and control strategy, library construction strategies, and bioinformatics analysis processes among different studies, which weakens the comparability of research results. Finally, most of the existing conclusions are limited to differential expression analysis, functional enrichment, and regulatory network inference, lacking in-depth causal experimental verification. Future research urgently needs to further expand the types of tissues and environmental stress models (such as altitude gradients, seasonal nutritional fluctuations, hypoxia/cold stress models, etc.), and on this basis, introduce single-cell RNA sequencing, spatial transcriptomics, and standardized experimental verification strategies, in order to construct a higher-resolution, more mechanism-oriented multi-dimensional spatiotemporal circRNA atlas of yaks.

### 4.2. circRNA–ceRNA Regulatory Axes Related to Adipogenesis and IMF Deposition

Based on the construction and differential analysis of circRNA expression profiles in yaks, some studies have further conducted systematic functional validations on the molecular mechanisms of adipogenesis and IMF deposition. These studies mainly focus on the “microRNA sponge” or “competitive endogenous RNA (ceRNA)” regulatory mechanism: that is, circRNA competes with microRNA (miRNA) for binding, thereby weakening the inhibitory effect of miRNA on the expression of target messenger RNA (mRNA), and subsequently regulating key phenotypes such as adipocyte proliferation, differentiation, and lipid deposition. According to the completeness of the evidence chain, the related studies can be summarized as experimentally verified representative regulatory axes and candidate regulatory modules predicted based on network analysis.

#### 4.2.1. Experimentally Verified Representative Regulatory Axes

**circCWC22–miR-3059-x–HMGCL Axis:** Negatively Regulates Adipogenesis and IMF Deposition. In the comparison experiment of high and low IMF content in yaks, circCWC22 was significantly upregulated in the low-IMF group, accompanied by downregulation of miR-3059-x and upregulation of HMGCL. This expression pattern suggests that this axis may be involved in the negative regulation of IMF deposition. The study confirmed the circular structure of circCWC22 through RNase R resistance detection and Sanger sequencing, and verified the specific interaction among circCWC22, miR-3059-x, and HMGCL through dual-luciferase reporter gene experiments and RNA immunoprecipitation experiments. Cell function intervention experiments showed that overexpression of circCWC22 significantly inhibited the proliferation and differentiation of adipocytes, resulting in weakened oil red O staining intensity and decreased triglyceride content, while downregulating the expression of adipogenesis-related genes such as PPARγ, C/EBPα, FASN, and SREBP1. Conversely, knockdown of circCWC22 had a promoting effect, consistently supporting the inhibitory effect of this axis in regulating adipogenesis in yaks.

**SE-circRHOQ–miR-5093–IRF5 Axis:** Inhibits Adipocyte Differentiation and Lipid Deposition. The super-enhancer-associated circRNA (SE-circRHOQ) has been confirmed to be involved in the regulation of IMF deposition in yaks. The study confirmed the circular feature and cellular localization of SE-circRHOQ through RNase R treatment, Sanger sequencing, and fluorescence in situ hybridization. Through dual-luciferase, AGO2 immunoprecipitation, and RNA pull-down experiments, the targeted relationships between SE-circRHOQ and miR-5093, as well as between miR-5093 and the target gene IRF5, were verified. Phenotypic analysis showed that overexpression of SE-circRHOQ reduced oil red O staining intensity and triglyceride accumulation, while inhibiting the expression of the key adipogenic gene PPARγ. Functional rescue experiments further confirmed this regulatory chain. Although the correlation direction of this axis may be affected by tissue heterogeneity in different populations, the molecular interaction and cell experiment data consistently support its inhibitory effect on adipocyte differentiation.

#### 4.2.2. Mechanism-Predicted Candidate Axes

**circ_0001610–miR-423-5p–SLC7A11 Axis:** Suggests that the feeding system may affect muscle phenotype through the ferroptosis pathway. In the study of yak longissimus dorsi muscle under different feeding systems, the circ_0001610–miR-423-5p–SLC7A11 axis was identified as a core regulatory module. This axis is closely related to SLC7A11-mediated redox homeostasis and ferroptosis, and is associated with phenotypes such as apoptosis. This suggests that nutritional conditions and environmental changes may regulate the homeostasis of muscle tissue through the ceRNA network, but causal validation at the cellular and animal levels is still needed.

#### 4.2.3. Regulatory Network Prediction and Cross-Tissue Integration Analysis

**circHIPK3–miR-124-3p–STAT3 axis:** As a candidate regulatory node related to IMF deposition. In the whole transcriptome study of muscle and fat paired tissues, researchers revealed a large-scale ceRNA regulatory network. Among them, the circHIPK3–miR-124-3p–STAT3 axis has attracted much attention due to its involvement in core processes such as the Wnt and β-catenin signaling pathways, cell proliferation, and apoptosis resistance. The subsequent research focus on such candidate axes is to supplement direct evidence of molecular interactions and improve the detection of phenotypes related to fat differentiation.

In summary, research on yak fat production can be divided into three levels: differential expression screening, structural stability verification, and causal elucidation of the complete regulatory mechanism. Future research should prioritize IMF traits directly related to meat quality and introduce single-cell resolution technology to reduce the interference of tissue heterogeneity.

### 4.3. Key Biological Processes and Multidimensional Mechanisms Mediated by circRNA: Exploration of Pathway Commonalities and Non-ceRNA Modes

**Figure 1:**
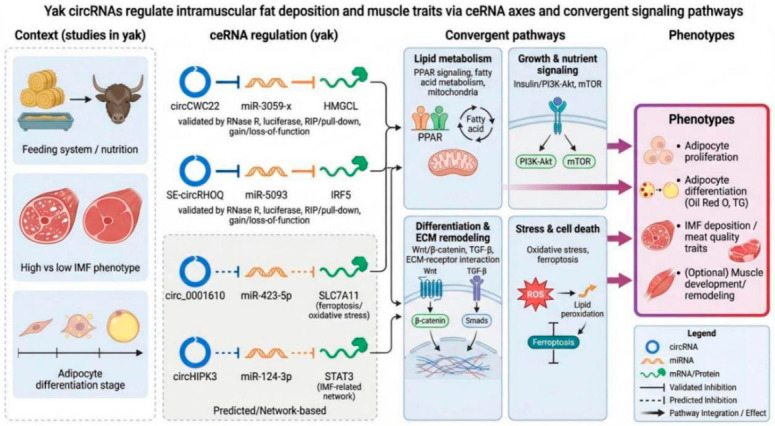
Integrative Mechanism of the CircRNA–ceRNA Regulatory Network and Downstream Convergent Pathways in Yaks and their Roles in IMF Deposition and Muscle Phenotypes. This figure systematically reviews the current molecular evidence system regarding the involvement of circRNAs in regulating IMF deposition and skeletal muscle development in yaks. The network architecture is divided from left to right into: physiological background and research models (including feeding patterns, nutritional interventions, phenotypic differences, and cell differentiation timelines), the post-transcriptional regulation layer (ceRNA network), and the downstream signaling pathway convergence layer. In the post-transcriptional regulation layer, the two representative regulatory axes, circCWC22–miR-3059-x–HMGCL and SE-circRHOQ–miR-5093–IRF5, clearly demonstrate the classic molecular mechanism of circRNAs functioning as “miRNA sponges” and reversing the inhibition of target gene expression; their multi-dimensional verification system includes molecular structure identification (RNase R resistance), target binding confirmation (dual luciferase, RIP, and RNA pull-down), and causal functional perturbation (overexpression and knockdown intervention) [68,69]. Meanwhile, the figure marks circ_0001610–miR-423-5p–SLC7A11 and other candidate bioinformatics prediction modules that need to be verified with dashed lines. In the convergence layer of signaling pathways, these upstream post-transcriptional regulatory events ultimately converge biochemical effects on four core functional modules: lipid metabolism network (PPAR and fatty acid metabolism), growth and nutrient sensing hub (insulin/PI3K-Akt and mTOR pathways), cell differentiation and extracellular matrix microenvironment remodeling (Wnt/β-catenin and TGF-β cascades), and stress response and cell fate determination (oxidative stress and ferroptosis) [60,66]. These convergent signaling cascades synergistically drive the proliferation and differentiation potential of adipocytes, as verified by Oil Red O lipid droplet staining (representative images) and triglyceride quantification, and profoundly influence the efficiency of IMF deposition, meat quality traits, and potential skeletal muscle morphological remodeling. Created with BioRender.

In the study of circRNA in yaks, although the current focus is mainly on the construction of ceRNA networks, the functional enrichment results across tissues and studies show that the regulation of circRNA in several key biological processes exhibits significant convergence. These regulatory nodes ultimately point to four major modules: lipid metabolism, nutrient signaling, structural remodeling, and stress and cell fate determination (Figure 1).

(i) Lipid Metabolism and Energy Homeostasis: Centered on PPAR, fatty acid metabolism, and AMPK pathways. Whole transcriptome studies related to the differences in IMF deposition in yaks have shown that the differentially expressed genes are significantly enriched in the AMPK signaling pathway, PPAR signaling pathway, and fatty acid biosynthesis process. This indicates that the influence of the circRNA regulatory network on IMF is mainly achieved by regulating the transcriptional program of fat generation and lipid metabolism pathways.
(ii) Growth and Nutrient Signaling: Involving the coordinated action of PI3K-Akt, MAPK, and cGMP-PKG pathways. Studies have shown that differentially expressed circRNAs in the longissimus dorsi muscle tissue are mainly associated with growth factor perception and metabolic execution processes. This suggests that the formation of IMF is not a single lipid deposition event, but a comprehensive process regulated by the coordinated action of nutrient sensing signals, providing a logical basis at the pathway level for understanding the differences in meat quality under different feeding systems.
(iii) Tissue Structure and Extracellular Matrix Remodeling: Simultaneous enrichment of muscle development-related items. In comparative studies of different feeding systems, functional enrichment items point to processes such as muscle organ development, myofibril assembly, and extracellular matrix receptor interaction. This indicates that circRNA not only affects lipid content but may also indirectly influence meat tenderness and shear force by regulating myofibril structure assembly and cell adhesion.
(iv) Stress and Cell Death: Regulatory entry points related to oxidative stress and ferroptosis. Some studies have proposed the circ_0001610–miR-423-5p–SLC7A11 axis, extending the regulatory perspective to the processes of cellular antioxidant defense and ferroptosis sensitivity. Considering the yak’s high dependence on redox homeostasis in extreme high-altitude environments, this direction provides new insights into how environmental stress shapes muscle tissue homeostasis.

In addition to the classic ceRNA regulatory mode, studies in general biology have demonstrated that circRNA can also function through non-classical mechanisms such as interacting with RNA-binding proteins, translating into functional peptides, and regulating transcription or splicing in the nucleus.

### 4.4. Overview of the miRNA Research Map and Experimental Grouping and Control Strategy in Yaks

As one of the non-coding RNAs that entered the systematic research field relatively early, research on miRNAs has generated a wealth of expression profile data and functional validation evidence in various tissues, environments, and developmental stages of yaks [70,71,72]. Existing literature indicates that the research materials of yak miRNA not only cover basic tissues such as testis, ovary, follicle, corpus luteum, skeletal muscle, and fat, but also extend to the heart, lungs, and milk-derived exosomes closely related to high-altitude adaptation. In terms of experimental grouping, it includes multiple aspects such as growth and development stages (e.g., comparison between juvenile and adult individuals), altitude gradients and hypoxia exposure, feeding and nutritional conditions, reproductive seasons and reproductive health status (e.g., differences between healthy and atretic follicles), and phenotypes of distant hybrid offspring (e.g., the mechanism of male sterility in yak-donkey hybrids) [70]. Through the combined analysis of small RNA sequencing (sRNA-seq) and whole transcriptome, researchers have successfully identified a large number of known and novel miRNAs in different tissues. Although the number of differentially expressed miRNAs identified in different studies varies, most studies have verified the core molecules through real-time quantitative PCR (RT-qPCR), providing a reliable candidate set for subsequent mechanism analysis [71,72].

Looking at the evolution of research themes, the research paradigm in the field of yak miRNA is undergoing a profound transformation from “expression profile mapping” to “regulatory mechanism axis analysis”: early work mainly focused on tissue-specific and stage-specific differential expression analysis and functional pathway enrichment inference (such as GO and KEGG); in recent years, experimental verification at the molecular target interaction and cellular phenotype levels has been gradually strengthened [73]. Particularly in the research on high-altitude adaptation and hypoxia response, miRNA has been repeatedly confirmed to be closely related to the hypoxia-inducible factor (HIF) pathway, PI3K-Akt and MAPK signaling cascades, and the apoptosis regulatory network [74]. This suggests that miRNA is likely to be a key regulatory hub connecting “environmental hypoxia stress” and “maintenance of tissue homeostasis in the body”. At the same time, in the field of fat generation and IMF deposition, researchers have also constructed multiple miRNA–target gene regulatory axes with high evidence chain integrity, such as those related to SIRT and FoxO1 proteins, and Toll-like receptor (TLR) signaling pathways, providing clear molecular clues for understanding the formation mechanism of yaks’ meat quality traits [75,76].

### 4.5. Yak miRNA Regulation and High-Altitude Hypoxia and Cardiopulmonary Adaptation: From Regulatory Networks to Elucidation of the Complete Regulatory Mechanism

**Figure 2:**
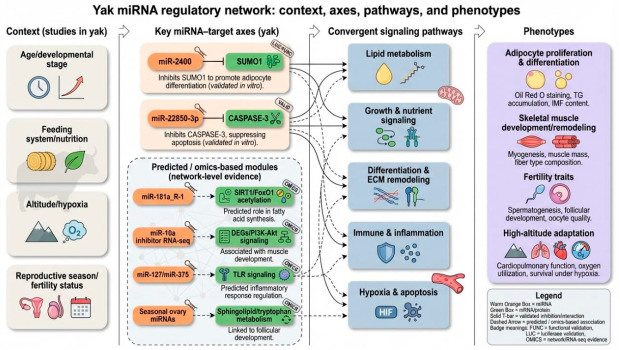
Yak miRNA Regulatory Network Map: Connecting Biological Contexts, Verified Regulatory Axes, Convergent Pathways and Phenotypic Traits. This figure systematically summarizes the following core elements [74,77,78,79,80,81]: (i) major biological contexts (age/development, nutrition, altitude/hypoxia, reproductive status); (ii) experimentally verified yak miRNA-target inhibitory regulatory axes; (iii) omics/network modules supported by differential expression and pathway enrichment analysis. Solid lines indicate experimentally verified direct inhibitory/interaction relationships (such as reporter gene detection and functional readout validation), while dashed lines represent predictive or omics-inferred associations. Convergent signaling pathways include lipid metabolism, growth and nutrient signaling (PI3K-Akt/MAPK/mTOR–insulin pathway), differentiation and extracellular matrix remodeling, immune and inflammatory responses, and hypoxia/apoptosis processes, which ultimately relate to phenotypic traits such as IMF deposition, skeletal muscle remodeling, reproductive traits, and high-altitude adaptation. Created with BioRender.

High-altitude hypoxia is the core environmental pressure driving the adaptive evolution of yaks, with its far-reaching impacts involving multiple physiological levels such as energy metabolism reprogramming, inflammatory response regulation, extracellular matrix (ECM) remodeling, and hypoxia-mediated apoptosis. Current evidence indicates that yak miRNAs can participate in the above processes through multiple convergent signaling pathways and ultimately produce significant effects on phenotypes such as IMF deposition, skeletal muscle structure remodeling, reproductive performance, and adaptation to high-altitude hypoxia (Figure 2). Notably, although the number of regulatory axes with complete evidence chains of “target binding validation” and “phenotypic function readout” in current yak miRNA research is still limited, some representative studies have successfully constructed a strict mechanistic closed loop.

In the hypoxia and apoptosis regulation module, miR-2285o-3p has been confirmed to directly target and inhibit CASPASE-3 expression, mediating anti-apoptotic responses that support yaks’ survival under low-oxygen conditions. Functional evidence indicates that its upregulation attenuates hypoxia-induced apoptosis and tissue damage, suggesting that miR-2285o-3p exerts cytoprotective effects by targeting the caspase cascade, representing a key molecular adaptation to high-altitude environments (Table 2).

In addition, in the lipid metabolism and tissue remodeling module, the miR-2400–SUMO1 regulatory axis constitutes another highly complete verification example. miR-2400 directly targets SUMO1 to modulate preadipocyte proliferation and differentiation. By inhibiting SUMO1 expression, this miRNA influences adipocyte fate decisions, thereby affecting IMF deposition and associated meat quality traits, and providing direct functional evidence for ceRNA-mediated regulation of yak adipogenesis (Table 2). From the perspective of the global regulatory network, the above strictly verified regulatory axes show a high degree of intersection and convergence with core pathways such as the PI3K-Akt, MAPK, and mTOR cascades, PPAR-mediated lipid metabolism networks, extracellular matrix remodeling, and immune inflammation (Figure 2). This not only provides theoretical support for understanding the formation of complex traits but also points out the exploration direction for subsequently condensing extensive “omics association data” into precise “core mechanism targets”.

In summary, research in the field of yak miRNAs is steadily advancing from primary expression profiling and pathway enrichment analysis to in-depth molecular mechanism validation. However, in the exploration of hypoxia tolerance and cardiopulmonary system adaptability, current research still urgently needs to rely on yak primary cell or tissue models to supplement more direct evidence of targeted interactions (such as 3′UTR luciferase reporter systems, RNA immunoprecipitation or cross-linking immunoprecipitation, etc.). At the same time, researchers must improve the cell function phenotype detection system highly related to hypoxia stress, thereby comprehensively enhancing the scientific explanatory power and independent repeatability of the existing post-transcriptional regulatory network.

### 4.6. Convergent Features of Cross-Organ Pathways: Immune and Metabolic Coupling of HIF-1α, PI3K-Akt, and MAPK Signaling Networks

High-altitude hypoxia is a key environmental pressure driving the long-term adaptive evolution of yaks, shaping organismal phenotypes through coordinated responses across multiple tissues and signaling pathways. Current transcriptomic and network inference analyses have revealed coordinated expression changes between the core HIF-1α hypoxia response module and pathways such as PI3K-Akt, MAPK, immune-inflammatory networks, and metabolic regulators, forming a convergent framework that underpins yaks’ high-altitude adaptation, as summarized in Figure 2. For instance, in a comparative transcriptomic study of the spleen between yaks and cattle, HIF-1α and its related hypoxia-inducible factors showed significant differential expression, suggesting that the hypoxia response in immune organs has distinct species-specific and altitude-adaptive characteristics, thereby providing an important theoretical basis for the “hypoxia and immunity” interactive regulatory mechanism [82]. Additionally, transcriptomic analysis of yaks and cattle pulmonary artery smooth muscle cells under different oxygen concentration gradients also indicated that the expression fluctuations of hypoxia-related metabolic regulatory factors (such as the PDK1-related pathway) occurred simultaneously with the adaptive phenotypic changes of the cells. This further confirmed that the hypoxia response in the cardiopulmonary vascular system is a complex adaptation process driven by metabolic reprogramming and signal network remodeling [83].

At the microRNA (miRNA) regulatory level, multiple studies based on the standard analysis paradigm of “expression difference screening, target gene prediction, and functional pathway enrichment” have proposed that the PI3K-Akt and MAPK signaling cascades, as well as the immune and metabolic interaction network, may be deeply involved in the high-altitude adaptation and regulation of key productive traits of yaks. For example, targeted inhibition of miR-10a in cell models combined with RNA sequencing analysis revealed that the PI3K-Akt signaling pathway likely serves as a core convergence node, accompanied by the expression reprogramming of genes involved in growth and development, lipid metabolism, and immune response, such as IGF2, FABP4, and IFNB1 [79]. In the immune and inflammatory module, miR-127 and miR-375 have been confirmed to be closely associated with the Toll-like receptor (TLR) signaling pathway, effectively regulating the expression of key factors in inflammatory and immune responses, such as IL6 and IFNB1. This finding strongly suggests that miRNAs play a crucial bridging role in coupling innate immune signals with the local metabolic state of the organism [80]. In reproductive system research, the seasonal differential expression of miRNAs in ovarian tissue is highly correlated with the significant enrichment of sphingolipid and tryptophan metabolic pathways, indicating that the dynamic fluctuations of metabolic networks may be involved in a coordinated mechanism with the regulation of reproductive endocrine function and seasonal reproductive phenotypes [81]. Moreover, existing studies have clarified that miR-181a_R-1 can interfere with the metabolic and differentiation processes of cells by mediating the acetylation pathway of SIRT1 and FoxO1 proteins, providing a strong supplementary example for the intertwined network of miRNA regulation of energy metabolism and cell differentiation programs [74].

Although existing studies have revealed multiple highly potential network interaction relationships, it must be pointed out that most current conclusions are still based on omics association analysis or transcriptome differential responses after gene knockdown. Compared with a rigorous mechanistic closed loop that includes direct target binding verification and in vitro functional phenotype determination, there is still a certain gap in the strength of evidence. In light of this, future research should prioritize focusing on the repeatedly emerging core convergence nodes mentioned above (including the HIF-1α transcriptional complex, and PI3K-Akt and MAPK signaling pathways, as well as TLR-mediated immune and metabolic networks). In terms of experimental design, it is recommended to rely on in vitro models of primary immune cells, vascular endothelial cells, adipocytes or skeletal muscle cells of yaks, and apply standardized hypoxic environment stimulation to systematically advance the target validation of candidate miRNAs (such as conducting 3′UTR dual-luciferase reporter gene experiments and RNA immunoprecipitation analysis, etc.). At the same time, it is urgent to simultaneously improve the quantitative determination system of key phenotypes (covering the release level of inflammatory factors, dynamic changes in metabolic flux, and precise assessment of cell differentiation, survival and apoptosis rates). Only in this way can the macroscopic omics pathway convergence framework be truly transformed into an independently reproducible and clearly causal core regulatory axis.

### 4.7. Key Methodological Challenges and Future Directions in Yak miRNA Research

Current research on yak microRNAs (miRNAs) has provided important molecular clues for understanding the regulatory mechanisms of high-altitude adaptation and related economic traits. However, from a broader perspective, the overall research is still highly dependent on differential expression screening and pathway enrichment analysis, generally constrained by the bottleneck of “redundant association evidence but lacking closed-loop mechanisms.” Specifically, the field currently faces two core challenges: First, there is significant heterogeneity among different studies in terms of tissue sampling sites, sampling seasons, altitude gradients, animal ages, and physiological states, which can easily lead to the misinterpretation of environmental or sampling biases as true biological regulatory differences; second, the validation of regulatory relationships between a large number of candidate miRNAs and target genes is mostly limited to bioinformatics algorithm predictions or transcriptional-level responses, severely lacking 3′UTR dual-luciferase wild-type and mutant reporter gene experiments, AGO2 immunoprecipitation (RIP), cross-linking immunoprecipitation (CLIP), Western blot verification, and cell or in vivo phenotypic readouts highly consistent with target traits. This directly limits the independent reproducibility and deep biological interpretability of research results.

To comprehensively enhance the scientific reliability, horizontal comparability, and application potential of yak miRNA research, this paper suggests establishing a standardized experimental verification roadmap for this field, which can be focused on the following three dimensions:

(i) Enhancing reliability: Promoting the research paradigm from “omics enrichment inference” to “causal mechanism validation.” Future research should prioritize focusing on core convergent modules that repeatedly appear in multiple omics data (such as hypoxia response, coupling of immune inflammation and metabolic networks, lipid metabolism, and tissue structure remodeling), and screen for high-confidence candidate miRNAs. On this basis, it is necessary to strictly construct a complete causal evidence chain covering “transcript expression changes–target binding validation–changes in target protein and metabolic flux–final phenotypic readout.” In the design of experimental studies, particularly when establishing in vitro cell models, it is crucial to standardize key parameters. This includes clearly defining the experimental grouping strategy (such as tissue sampling sites, seasonal characteristics, altitude information) and the biological attributes of the source animals (age, gender, and physiological cycle). Such standardization is essential to strictly control for potential confounding factors and thereby confirm the uniqueness and causality of miRNA regulatory relationships [84].

(ii) Strengthening comparability: Establishing a standardized system for sample metadata collection and sequencing analysis processes. At the beginning of the experimental design, researchers should clearly define and as much as possible unify key parameters such as tissue sampling sites, seasonal characteristics, altitude information, and the age, gender, and physiological cycle of experimental animals, and strictly correct for potential confounding factors in subsequent statistical models. In the small RNA sequencing (sRNA-seq) and real-time fluorescent quantitative PCR verification stages, it is necessary to report in detail the library quality control standards, data normalization strategies (such as the scientific selection of reference genes or the introduction of exogenous spike-in RNA), batch effect elimination algorithms, and specific effect size indicators. In addition, the field should actively advocate independent replication studies across cohorts, promote the open-source sharing of raw sequencing data and analysis codes, and fundamentally enhance the reproducibility of post-transcriptional regulatory maps [85].

(iii) Promoting usability: Achieving the transformation from “basic candidate molecule list” to “clinically translatable diagnostic panel.” If the goal is to develop specific miRNAs as molecular markers for high-altitude adaptation or as breeding assistance evaluation indicators for superior traits, strict validation must be conducted in independent large-scale animal populations. This process must adhere to the international reporting standards for biomarker prediction models, clearly evaluate and publicly disclose their predictive performance (such as the area under the receiver operating characteristic curve AUC, sensitivity and specificity) and their stability in complex backgrounds (such as across different breeding seasons, geographical regions and dietary management conditions), thereby minimizing the risk of selective reporting bias and false positives [86]. At the same time, future mechanism dissections should deeply integrate multi-omics platforms, single-cell transcriptome sequencing and spatial transcriptomics technologies to precisely identify the specific cell population sources of key regulatory axes and their communication pathways in the tissue microenvironment, avoiding over-inferences due to mixed tissue sequencing.

In summary, the core incremental space for future research on miRNA in yaks lies in solidifying the complete closed loop of the mechanism evidence chain, promoting cross-population cohort replication and validation, and fully implementing the standardization of experimental and analytical processes. Only in this way can the current massive but fragmented “regulatory network association data” be substantially transformed into mechanism-explainable, cross-study comparable, and industrially applicable molecular regulatory rules and breeding assistance tools.

## 5. Yak lncRNA: Regulatory Networks, Mechanism Evidence and Research Routes

Building on the systematic review of the research progress and common challenges of miRNA in yaks, as well as the evidence chain roadmap constructed based on “standardization of sample analysis, strict data quality control, confirmation of targeted mechanisms and normalization of cohort validation”, this chapter shifts its focus to long non-coding RNA (lncRNA). Compared with miRNA, lncRNA exhibits more significant tissue and spatiotemporal expression specificity, and its molecular mechanisms of function are more complex: it can precisely regulate the expression of adjacent and distant genes through cis or trans pathways, can act as a competitive endogenous RNA to form lncRNA–miRNA–mRNA cascade interaction networks, and can even directly bind to chromatin and transcription factors, deeply participating in epigenetic reprogramming. In recent years, thanks to the mapping of high-quality reference genome maps of yaks and the wide application of long-read sequencing and single-cell transcriptomics and other cutting-edge technologies, lncRNA candidate regulatory axes related to hypoxia adaptation, skeletal muscle development, and lipid and energy metabolism have been continuously discovered. However, the lack of unified transcript annotation standards, the absence of comparability between cross-cohort studies, and the weakness of deep mechanism verification remain the key bottlenecks restricting breakthroughs in this field. In view of this, this chapter aims to systematically analyze the current research status and technological evolution path of lncRNA in yaks, distill its core action patterns and regulatory network architecture, focus on summarizing representative achievements in hypoxia adaptation, muscle development and lipid metabolism, and propose standardized verification strategies aimed at achieving elucidation of the complete regulatory mechanism and translational application.

### 5.1. Research Overview and Development Status of Yak lncRNA

With the continuous optimization of yak genome annotation and the rapid development of high-throughput transcriptome sequencing, research on yak lncRNAs has moved far beyond simple screening of differentially expressed transcripts, and advanced into in-depth exploration of spatiotemporal expression patterns and complex regulatory networks. A large number of uncharacterized lncRNA transcripts have been screened across multiple yak tissues and altitude environments, laying a molecular foundation for exploring altitude-related physiological characteristics [87]. By deeply integrating long-read transcriptome assembly technology and single-cell transcriptomics data, researchers have further revealed at the level of cell subtypes and tissue microstructures that lung-specific lncRNA is highly likely to deeply mediate key high-altitude adaptation processes such as pulmonary vascular remodeling [88]. In terms of the exploration of molecular mechanisms, some studies have focused on the physiological background of hypoxia adaptation and innovatively introduced cell stress models such as ferroptosis, relying on the co-expression network of lncRNA and mRNA to screen core driver elements, which clearly indicates that lncRNA widely penetrates and regulates the body’s oxidative stress response and energy metabolism homeostasis [89].

From the current distribution of research hotspots, the adaptive reprogramming of skeletal muscle growth and development and energy metabolism constitute the two core pillars of yak lncRNA research. For example, high-resolution temporal transcriptomics evidence indicates that in the development of the longissimus dorsi muscle and IMF deposition in yaks, lncRNA shows highly stage-specific dynamic expression patterns and network topological features [90]; at the same time, some scholars have conducted in-depth in vitro functional verification of specific key lncRNAs, confirming that they can precisely intervene in the expression of core genes in myoblast differentiation through the classic competitive endogenous RNA interaction model [91]. In the study of core metabolic organs, a comprehensive analysis of the yaks liver transcriptome across different growth and development stages has pointed out that specific lncRNA communities are deeply involved in maintaining the underlying metabolic pathways of the liver and responding to environmental stress [92]; in addition, in the macroscopic background of high-altitude adaptive evolution, a series of lncRNA candidate molecules highly enriched in energy metabolism pathways have been successively identified, providing highly valuable molecular targets for subsequent targeted analysis of lipid metabolism flow and oxidative energy supply mechanisms [93].

However, it must be acknowledged that the current yak lncRNA field still faces severe challenges such as highly fragmented transcript annotation and low comparability of research results across different cohorts. This is mainly attributed to the high heterogeneity in the underlying methodologies among different research teams, specifically including library construction strategies (such as the use of poly(A) tail enrichment methods and ribosomal RNA depletion methods, as well as whether to retain strand-specific information, etc.), transcript assembly algorithms, expression abundance cutoff thresholds, and the combination differences in coding potential assessment tools. These technical barriers directly result in a low overlap of lncRNA catalogs identified in independent studies, severely weakening the secondary utilization value of sequencing data. To reduce the false positive rate from the source and significantly enhance the logical consistency of cross-study results, the current methodological consensus advocates the deep integration of multi-dimensional matrices such as the intrinsic base composition, higher-order structural features, and physicochemical properties of sequences to implement precise identification and machine learning classification modeling of lncRNA [94]. Meanwhile, it is called for that the industry widely adopt highly integrated bioinformatics analysis pipelines to establish absolute standardization and reproducibility in the processes of transcript screening, quality control filtering, and fine annotation [95]. In summary, although the field of yak lncRNA has accumulated considerable data assets and preliminary evidence on key issues such as hypoxia adaptation, skeletal muscle evolution, and metabolic homeostasis regulation, the future breakthrough lies in the vigorous promotion of unified annotation standards, the acceleration of independent replication across cohorts, and the breakthrough in the in-depth confirmation of molecular mechanisms. Only in this way can the research field truly drive a leap from shallow “network association inference” to “causal mechanism analysis” and “functional molecular marker transformation”.

### 5.2. Regulatory Modes and Network Architecture of Yak lncRNA

Unlike the relatively clear base sequence complementarity and target inhibition pathways of microRNAs (miRNAs), long non-coding RNAs (lncRNAs) function in a more complex manner. Their biological roles rely heavily on spatiotemporal expression patterns, subcellular localization and specific structural domains, as well as intricate interactions with DNA, RNA and proteins. Therefore, lncRNAs often exhibit more significant specificity in different tissues and developmental stages [96]. Currently, it is widely recognized in the academic community that lncRNAs can specifically recruit or isolate downstream regulatory factors through core modes such as “guiding molecules”, “molecular scaffolds”, and “molecular decoys”, thereby deeply participating in transcriptional regulation, maintenance of chromatin higher-order structure, and post-transcriptional processing and translation regulation. At the same time, the in-depth analysis of lncRNA spatial structure and interactome provides key technical means and evidence support for clarifying their molecular mechanisms [96]. In the field of yak-related research, existing functional evidence mostly comes from the construction of transcriptome-level expression profiles, the mining of co-expression modules, and the inference of pathway enrichment, but it is currently steadily advancing towards the in-depth direction of “from candidate target axis screening to mechanism closed-loop verification” [89,93,97].

Focusing on the regulatory pathways of yak lncRNA, its regulatory modes can mainly be classified into cis and trans categories [96,97]. Among them, cis regulation usually takes the physical proximity in the genome as the inference clue: if a specific lncRNA and its physically adjacent protein-coding gene show highly consistent or significantly correlated expression fluctuations under specific physiological conditions or experimental treatments, researchers often conduct functional pathway enrichment analysis of the adjacent genes, and then propose the scientific hypothesis that “this lncRNA may regulate the expression of adjacent genes in a cis manner by interfering with the assembly of local transcriptional complexes or altering the chromatin open state”. In contrast, the analysis of trans regulation relies more on the algorithmic screening of co-expression networks or co-expression modules: if a certain lncRNA and a distant gene group construct an extremely stable co-expression topological module, and the biological function enrichment results of this module are highly consistent with the macroscopic phenotype, this lncRNA can be established as a priority candidate molecule for subsequent mechanism verification. It is necessary to solemnly point out that the genomic proximity relationship or statistical correlation itself is by no means equivalent to a biological causal relationship. Based on this, cutting-edge research strongly recommends that in the stage of inferring co-expression regulatory networks, strict measures must be taken to control potential false positive interferences (such as effectively correcting batch effects, avoiding tissue heterogeneity interferences, and eliminating false correlations caused by low expression abundance, etc.) [95], and independent validation datasets or multi-omics evidence should be actively introduced for cross-verification to effectively enhance the scientific reliability of mechanism inference.

At the level of regulatory network architecture, the competitive endogenous RNA (ceRNA) interaction model composed of lncRNA, miRNA, and targeted messenger RNA (mRNA) is one of the most mainstream strategies for currently resolving the functions of non-coding RNA in yaks. This strategy is particularly suitable for highly integrating massive differentially expressed transcripts and target genes into candidate regulatory hubs and establishing logical mappings with core pathways such as hypoxia response, skeletal muscle development, and lipid metabolism. However, the authenticity of the ceRNA regulatory mechanism is highly constrained by the absolute expression abundance of molecules, the co-localization characteristics of subcellular spatial positioning, and the biophysical accessibility of binding sites, among other prerequisites. If one merely relies on algorithmic predictions based on underlying sequences and seriously lacks experimental confirmation of expression trend coordination, dose-effect determination, or physical molecular interactions, it is very likely to lead to over-interpretation or misjudgment of the regulatory mechanism [98]. Therefore, when constructing large-scale ceRNA regulatory networks, it is strongly recommended to adhere to the core principle of “high-confidence database traceability combined with strict threshold screening”: the selected target axes should at least meet the requirements of co-expression in the same tissue or single-cell population, a negative correlation between miRNA and its targets that conforms to biological logic, and a high consistency between the underlying regulatory network function and macroscopic phenotypes; at the same time, it is necessary to integrate database cross-validation, support from previous literature, and in vitro experimental confirmation as much as possible to comprehensively consolidate the robustness of the conclusion [99]. Recent comparative experiments based on the gene expression profiles of bovine species have also shown that there is significant heterogeneity in the lncRNA expression profiles between muscle and fat tissues, which further warns that the specificity of tissues and cell types will profoundly constrain the construction of ceRNA networks and the boundaries of their biological interpretations [99].

**Figure 3:**
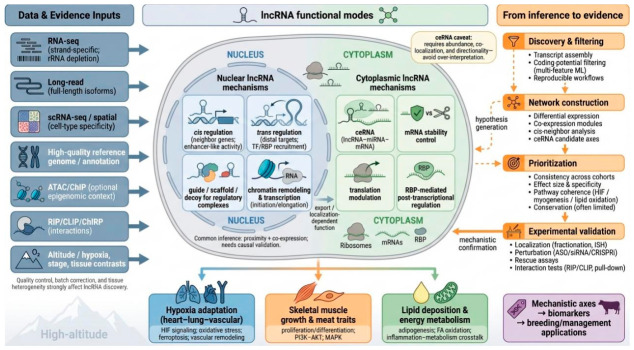
Functional Models, Regulatory Network Architectures and Systematic Research Processes of Long Non-coding RNAs (lncRNAs) in Yaks.

In summary, based on the existing data types and the overall research status quo, this article systematically distills the core functional patterns of lncRNA in yaks and the entire process from front-end bioinformatics inference to back-end experimental mechanism confirmation (Figure 3). In short, the research paradigm in the field of yak lncRNA can be highly summarized as a three-step strategy of “discovery to network inference and then causal validation”: first, relying on high-throughput RNA sequencing technology to accurately assemble transcripts from scratch and perform absolute quantification calculations, and introducing coding potential assessment algorithms that integrate multiple sequence features and complex models to purify the lncRNA candidate set (such as deploying machine learning frameworks or integrated standard workflows to minimize the false positive rate) [94,95]; second, comprehensively applying bioinformatics strategies such as differential expression analysis, co-expression clustering mining, cis-proximity target gene inference, and ceRNA topological network construction to precisely anchor core candidate regulatory axes, and closely focusing on macroscopic phenotypes such as high-altitude hypoxia adaptation, skeletal muscle growth and development, and lipid and energy metabolism for function-oriented screening [89,90,93]; finally, systematically conducting subcellular localization determination and molecular interaction confirmation at the cellular and in vivo levels (for example, clarifying the spatial distribution of lncRNA through nuclear–cytoplasmic separation technology combined with fluorescence in situ hybridization, establishing causal logical relationships through targeted knockdown, gene overexpression, and functional recovery experiments, and resolving complex physical interactions through molecular pull-down and co-immunoprecipitation experiments). Through the above rigorous process, the primary “transcriptional network associated signals” are ultimately substantially converged into core regulatory rules that are mechanistically interpretable and independently reproducible [96]. Following this theoretical framework, the following text will closely revolve around the three core themes of hypoxia adaptation, muscle development, and lipid metabolism, systematically summarizing the most representative evidence chains and core regulatory axes in the current research field of lncRNA in yaks [98].

This figure systematically integrates the core evidence sources in current yak lncRNA research (including second-generation transcriptome sequencing, third-generation long-read sequencing, single-cell and spatial transcriptomics, and genome annotation, as well as epigenomic and interactome detection technologies), and deeply reviews the biological functions of lncRNAs based on their subcellular spatial localization [96]. In the nucleus, lncRNAs mainly regulate the expression of adjacent genes through the *cis* mode, or act as “molecular scaffolds”, “guiding molecules” and “molecular decoys” to recruit regulatory complexes, and intervene in chromatin higher-order structure and transcription initiation processes through the *trans* mode [97,99]. In the cytoplasm, lncRNAs are widely involved in post-transcriptional regulation, including constructing ceRNA networks (lncRNA–miRNA–mRNA interactions), regulating mRNA stability and interfering with protein translation. The right panel of the figure highlights a complete standardized research path from “transcript identification, network construction, candidate axis priority screening to mechanism closed-loop verification”, clearly defining the logical boundary between speculative associations and causal experimental verification [98]. This process closely aligns with key application areas in yak research, including adaptation to hypoxic environments, skeletal muscle growth and development, and lipid and energy metabolism remodeling, aiming to promote the transformation of related achievements into the resolution of core regulatory mechanisms and the development of molecular markers for superior traits.

### 5.3. Core Areas of Yak lncRNA Research: Hypoxia Adaptation, Skeletal Muscle Development, and Lipid Metabolism Reprogramming

Yaks have long thrived in the extreme ecological environment of high altitudes and low oxygen levels. Research on lncRNAs in this species has mainly focused on key biological processes such as hypoxia signaling (e.g., the cascade reaction mediated by hypoxia-inducible factor, HIF), adaptive remodeling of the cardiopulmonary system, immune inflammation, and oxidative stress. In cross-species and cross-strain transcriptomic comparisons of muscle tissues, researchers have successfully identified a large number of novel and significantly differentially expressed lncRNAs in yak gluteus maximus. Through the screening of *cis*-adjacent genes (±100 kb regions) and *trans*-target prediction, a preliminary map of their potential target genes has been drawn. Functional pathway enrichment results suggest that these lncRNAs are likely to be deeply involved in the yak’s adaptation to high-altitude environments by precisely regulating energy metabolism and muscle contraction-related physiological processes. The expression trends of core candidate molecules have been highly consistently and independently verified through real-time fluorescent quantitative PCR technology [93]. At the organ system level, existing studies have compared the lung tissues of yaks at different altitudes and low-altitude control cattle, systematically identified the expression profiles of mRNA, lncRNA, and miRNA, and constructed a complex ceRNA topological regulatory network based on this. Differential expression analysis and biological function enrichment results reveal that the network nodes are highly enriched in core modules such as fatty acid metabolism, protein processing and modification, immune response, and cell cycle, thereby providing solid histological evidence for clarifying the molecular mechanism of the coupling of “cardiopulmonary system and hypoxia adaptation” [100]. At the in vitro cell model level, some studies have used yak coronary artery smooth muscle cells (CASMCs) as the research object and set up strict hypoxia (5% O_2_) and normoxia (21% O_2_) control groups. By combining CCK-8 cell viability assays and quantitative analysis of proliferating cell nuclear antigen (PCNA) protein, it was confirmed that hypoxia can significantly promote the abnormal proliferation of CASMCs, and simultaneously, a large number of transcripts that underwent systemic expression reprogramming (including 835 mRNAs, 285 lncRNAs, and 126 miRNAs) were identified. Data mining further indicated that multiple key signaling pathways such as TGF-β, MAPK, cAMP, mTOR, and PI3K-Akt constitute the core network of the hypoxia response. Based on this, researchers constructed a specific ceRNA interaction network and confirmed the dynamic response trends of core molecules through quantitative PCR [101]. Additionally, cutting-edge exploration has begun to integrate hypoxia stress signals with meat quality economic traits. For example, in the longissimus dorsi muscles of yaks with differentiated IMF content, researchers not only constructed an IMF-related ceRNA regulatory network but also, through functional perturbation experiments targeting HIF-1α, strongly suggested that hypoxia-related pathways may directly mediate lipid deposition and substrate metabolism reprogramming, thereby deeply coupling the two macroscopic phenotypes of “extreme environment adaptation” and “excellent economic traits” at the molecular level [102]. Overall, lncRNA research related to yak hypoxia adaptation is steadily advancing from the primary stage of “differential screening combined with enrichment inference” to the higher stage of “integration of network inference based on specific tissue and cellular contexts and in-depth functional verification”. Skeletal muscle growth and development and meat quality characteristics (especially IMF deposition) are the core issues that receive the most attention in lncRNA research on yak economic traits. High-resolution transcriptomic studies have precisely characterized the lncRNA expression landscape during the development of the longissimus dorsi muscle, and by combining target gene inference and co-expression network construction, have revealed the extensive roles of lncRNAs in regulating myogenesis, cell cycle operation, energy homeostasis, and lipid accumulation. For instance, in a longitudinal study of Tianzhu white yaks, by comparing the transcriptomes of the longissimus dorsi muscle at 6, 30, and 54 months of age, the core lncRNAs and their associated targets were significantly enriched in classic metabolic pathways such as AMPK, PI3K-Akt, and PPAR [90]. In a comparative sequence covering earlier developmental stages from fetal to adult, the *cis* and *trans* target genes of differentially expressed lncRNAs comprehensively covered core interconnected pathways such as *HIF-1*, *PI3K-Akt*, *AMPK*, *MAPK*, and apoptosis; simultaneously, the inclusion of key transcriptional regulatory factors such as *RTL1*, *IGF2*, *MEF2C*, and *PAX7* in highly co-expressed modules strongly suggests that this lncRNA community not only dominates the development program of yak muscle but also provides essential adaptive protection mechanisms for the postnatal high-altitude hypoxic environment [97]. Recently, in-depth analysis of the longissimus dorsi muscle of yaks and yak–cattle hybrids using Nanopore full-length transcriptome sequencing technology has further captured valuable complete transcript structures and alternative splicing events. This study not only systematically identified the lncRNA catalog but also, through pathway analysis, clearly indicated that MAPK and JAK-STAT cascades might be the core networks driving the muscle development differences in hybrid offspring; concurrent protein–protein interaction (PPI) analysis also suggested that specific alternative splicing subtypes of *TNNI2*-related transcripts might be the key factors determining the phenotypic differences in meat quality [103]. Notably, at the molecular mechanism level, a highly complete functional validation loop has emerged in this field: in the proliferation and differentiation model of primary yak myoblasts, Lnc-MEG8 exhibited significant dynamic redistribution characteristics between the nucleus and cytoplasm; through rigorous gene overexpression and targeted knockdown experiments, combined with multi-dimensional cell cycle and differentiation index evaluations (including PI staining, EdU incorporation detection, CCK-8 assay, and transcriptional quantification and Western blot analysis of *MYF5* and *MYOG*), it was conclusively demonstrated that Lnc-MEG8 promotes myoblast expansion and inhibits terminal differentiation. Subsequent series of confirmatory experiments, including the dual-luciferase reporter system, further revealed that miR-22-3p could directly interact with Lnc-MEG8 and the core target gene *RTL1*, perfectly interpreting the underlying molecular logic of the “Lnc-MEG8-miR-22-3p-*RTL1*” regulatory axis in driving myogenesis [91]. Thus, this topic has successfully established a mature research paradigm of “early omics discovery, candidate molecule selection to core target axis validation”; the future breakthrough point lies in vigorously promoting cross-population cohort replication and accumulating more systematic in vivo causal perturbation evidence to substantially enhance the translational application value of candidate lncRNA molecules in modern breeding. Research on lncRNAs related to lipid deposition and energy metabolism homeostasis in yaks has now comprehensively covered three core levels: IMF deposition dynamics, subcutaneous fat phenotype differentiation, and underlying metabolic regulation in the liver. The temporal evolution of the longissimus dorsi muscle has shown that the transcriptional abundance of lncRNAs during muscle development is highly dynamically coupled with the spatial deposition of IMF, and the differentially expressed mRNAs and lncRNAs are mainly concentrated in energy homeostasis hubs such as AMPK, PI3K-Akt, and PPAR, providing a clear roadmap for decoding the underlying transcriptional reprogramming during the IMF deposition process [90]. In a comparative study that closely mimics actual production models, scholars systematically evaluated the whole transcriptome response characteristics of subcutaneous fat in yaks under traditional grazing and intensive confinement feeding conditions, and successfully constructed a multi-level ceRNA topological network (a total of 677 differentially expressed mRNAs, 120 differentially expressed lncRNAs, 2216 differentially expressed circRNAs, and 15 differentially expressed miRNAs were identified). Based on the large-scale omics data, researchers not only identified the dominant pathways such as PPAR, PI3K-Akt, and cAMP, but also creatively proposed a potential key metabolic regulatory axis represented by the single core element TCONS00042948 and “TCONS00012083-bta-miR-2316-*MCAT*” and *NR4A3* [104]. As the absolute hub of energy metabolism and material conversion in the body, the liver also revealed a differentially expressed lncRNA community closely related to metabolism in the integrated transcriptome and co-expression network analysis across different growth stages. Bioinformatics inference confirmed that this community was widely involved in underlying biochemical processes such as lipid metabolism flow, collagen network remodeling, and transmembrane protein transport, and the age-dependent expression trajectories of some core lncRNAs have been independently verified experimentally [92]. It is crucial to note that there may be a hidden interactive regulatory network between the activation of the lipid deposition program and hypoxia stress signals: the aforementioned multi-dimensional omics and functional perturbation studies on individuals with different IMF contents (using HIF-1α gene overexpression and small interfering RNA targeted knockdown techniques) have initially confirmed that the hypoxia-inducible factor complex can directly reshape the lipid metabolism network and thereby determine the deposition efficiency of IMF [102]. In summary, this field has initially established a multi-organ joint evidence framework covering “skeletal muscle, adipose tissue to liver system”. However, to avoid long-term explanations of regulatory mechanisms remaining at the level of statistical correlation network inference, future research urgently needs to conduct more compelling experimental validations of the exact targets and causal effects of key lncRNAs through in vitro physical interaction assays and in vivo functional knockout models (Table 3).

### 5.4. Validation System and Application Transformation Path for Yak lncRNA Research

The current research on yak lncRNA has initially established a clear progressive research paradigm of “preliminary discovery, mechanism validation, and production application”. Specifically, this process begins with deep transcriptome sequencing of multiple tissues and developmental stages, screening candidate lncRNAs through differential expression analysis, and then combining cis and trans target gene prediction, biological function enrichment, and co-expression network analysis to initially identify a core set of molecules with research value [90,93,97,100]. Subsequently, in cell or tissue models that more closely simulate real physiological conditions, the research scope of candidate molecules is further narrowed down based on the competitive endogenous RNA (ceRNA) regulatory network and key signaling pathway framework [101,102]. Finally, through precise cell function intervention experiments and molecular interaction mechanism validation, a logical closed loop is completed from “statistical association signals” to “biological causal mechanisms” [91]. Meanwhile, the extensive application of long-read full-length transcriptome sequencing technologies such as Nanopore has significantly improved the resolution of transcript structure, isoforms, and alternative splicing events in the muscle tissues of yaks and their hybrid populations, providing a more reliable “reference coordinate system” for the precise annotation, quantitative analysis, and subsequent validation of lncRNAs, effectively reducing the uncertainty caused by traditional short-read assembly errors [103].

In the design of the validation route, this paper suggests standardizing the evidence chain system of lncRNA into four progressively advancing levels. The first is the expression and localization level, focusing on the tissue and cell type specificity, subcellular nuclear–cytoplasmic distribution characteristics, and spatiotemporal expression dynamics of the molecule. The second is the physical interaction relationship level, which acquires physical evidence of direct binding between lncRNA and miRNA, proteins, or genomic DNA through methods such as dual-luciferase reporter gene assays, RNA immunoprecipitation (RIP), and molecular pull-down experiments. The third is the causal perturbation level, which implements functional perturbations using gene overexpression, targeted knockdown intervention, or CRISPRi and CRISPRa technologies, and conducts rigorous rescue experiments (rescue assay). The fourth is the phenotypic readout level, covering quantitative indicators such as cell proliferation and differentiation rates, lipid droplet formation efficiency, and the expression levels of key marker genes and proteins. Currently, the field of yak lncRNA has produced highly valuable validation examples. For instance, research has confirmed that Lnc-MEG8 can significantly affect the proliferation and differentiation of myoblasts through the “Lnc-MEG8–miR-22-3p–RTL1” regulatory axis, and this work has constructed a complete validation loop through spatial localization, causal intervention, targeted detection, and phenotypic analysis [91]. Additionally, in the cross-research context of “hypoxia response and lipid deposition”, overexpression and interference experiments of HIF1α combined with adipogenic phenotype detection have provided decisive experimental support for the inference of transcriptional regulatory networks [102].

In terms of the application transformation route, the realization of the value of lncRNA in yak molecular breeding and precise breeding management can rely on the following three core paths. First, stable and repetitive candidate lncRNAs or their core regulatory modules should be associated with phenotypic data and genetic variations (such as expression quantitative trait loci, eQTL, or selection signals) of large-scale populations, and then developed into molecular markers for improving adaptability to high-altitude environments and meat quality traits. Secondly, it is necessary to deeply analyze the regulatory mechanisms of phenotypic plasticity mediated by feeding systems or environmental factors (such as the differences between grazing and stall feeding), and establish an interpretable chain from “environmental nutritional intervention, remodeling of the transcriptional regulatory network to changes in IMF deposition phenotype”, providing precise molecular intervention targets for optimizing the management strategies of yak breeding [104]. Finally, relying on the continuously improved full-length transcript annotation and isoform resources, the identified key regulatory axes should be extended to the splicing isoform and multi-level cascading regulatory network levels, significantly enhancing the robustness and transferability of candidate targets across different breeds [103].

In summary, the focus of future research on lncRNAs in yaks should continue to be on the replication of cross-population cohorts, in-depth in vivo experimental verification, and the accumulation of standardized data resources. Only by constructing a multi-dimensional rigorous evidence system can lncRNAs be substantially elevated from “mechanistically explainable candidate molecules” to “production-applicable molecular targets”, thereby providing a solid post-transcriptional regulatory theoretical basis for the technological innovation of plateau characteristic animal husbandry.

## 6. Summary and Outlook

Non-coding RNAs (ncRNAs) as core elements in post-transcriptional gene regulation are profoundly reshaping our understanding of the mechanisms underlying yaks’ adaptation to high-altitude environments and the formation of important economic traits. This article systematically reviews the biogenesis pathways and core regulatory patterns of three key ncRNAs: circular RNAs (circRNAs), microRNAs (miRNAs), and long non-coding RNAs (lncRNAs). By integrating the latest research progress in multiple tissues and organs, various growth and development stages, and multiple environmental contexts (including altitude gradients and hypoxia stress, different feeding management models, and differences in IMF deposition), this article comprehensively summarizes the current experimental evidence-supported core regulatory networks and the methodological bottlenecks that need to be overcome. Overall, yak ncRNA research has successfully transitioned from the initial stage of “transcriptional expression profiling and omics difference analysis” to the new stage of “complex network integration and molecular mechanism validation”. However, this field still shows significant imbalances: descriptive mapping sequencing is abundant, and bioinformatics-inferred networks are complex, but studies with a rigorous mechanism loop of “physical interaction confirmation, causal functional perturbation, and terminal phenotype readout” are still relatively scarce. Additionally, the high heterogeneity in sample cohort design, bioinformatics analysis pipelines, and experimental validation depth among different independent studies severely restricts the horizontal comparability of cross-study conclusions and the reusability of data assets.

In the circRNA field, existing studies have established relatively complete expression dynamic catalogs in yak skeletal muscle, adipose tissue, mammary glands, and male reproductive systems, and have established a representative research paradigm of “differential expression screening, ceRNA network inference, interaction mechanism validation, and functional phenotype readout” under the core theme of adipogenesis and IMF deposition. Particularly, the in-depth experimental confirmation of key regulatory axes such as circCWC22–miR-3059-x–HMGCL and SE-circRHOQ–miR-5093–IRF5 not only confirmed that circRNAs can intervene in adipocyte fate determination through the classic “miRNA sponge” mechanism but also revealed the high convergence of post-transcriptional regulation in core pathways such as lipid metabolism (PPAR and AMPK pathways), nutrient signal perception (PI3K-Akt, MAPK, and mTOR cascades), extracellular matrix remodeling, and cell stress and death (such as oxidative stress and ferroptosis). However, current research still overly focuses on the ceRNA model, and the exploration of non-classical mechanisms such as RNA-binding protein (RBP) interactions, nuclear transcription and alternative splicing regulation, and potential translation products of circRNAs is clearly insufficient. A more prominent shortcoming is that there is still a lack of spatiotemporal multi-dimensional maps of circRNAs in core driving organs for high-altitude hypoxia adaptation, such as the heart and lungs, which greatly limits the theoretical explanatory power of this molecule in understanding the mechanisms of adaptation to extreme environments. Currently, there are significant gaps in these areas in the study of yak circRNA. Future research should focus on the following directions:

1. Conduct systematic screening for RNA-binding protein interactions, such as using RNA immunoprecipitation sequencing (RIP-seq) technology;
2. Evaluate the translation potential of circRNA and obtain ribosome binding evidence, such as through polyribosome profiling or ribosome footprint sequencing (Ribo-seq) verification;
3. Deeply analyze the regulatory effects of nuclear-localized circRNA on host gene transcription, alternative splicing, and chromatin state.

By introducing subcellular localization stratified analysis, proteomics validation, and other experimental strategies, it will be helpful to construct a more complete and three-dimensional functional map of yak circRNA.

In the miRNA field, related research started earliest and covered the broadest tissue dimensions, especially in directions such as hypoxia response, anti-apoptotic protection, and the coupling regulation of immune inflammation and underlying metabolism, accumulating a vast amount of omics clues and pioneering the emergence of highly mechanism-closed evidence for strong logical target axes (such as miR-2285o-3p targeting and inhibiting CASPASE-3 to mediate hypoxia-induced apoptosis regulation; miR-2400 targeting SUMO1 precisely regulating the proliferation and differentiation programs of preadipocytes). Multi-dimensional pathway enrichment repeatedly points to the hypoxia-inducible factor (HIF)-mediated module and PI3K-Akt and MAPK signaling networks, revealing that miRNAs are highly likely to be the key regulatory hubs for achieving the cross-scale coupling from “environmental stress perception, maintenance of tissue homeostasis to macroscopic phenotypic output”. Looking ahead, the breakthroughs in this direction should no longer be about blindly expanding the list of differentially expressed molecules, but should focus on those network convergence nodes that repeatedly appear in cross-cohort omics, and strive to build a seamless evidence chain of “miRNA targeting, dynamic fluctuations of target proteins or metabolic fluxes, and final phenotypic readout”. At the same time, it is urgently necessary to conduct validation in in vitro models that highly simulate the real physiological conditions of the plateau (such as set hypoxia concentration gradients, specific inflammatory factor stimulation, and controlled energy supply environments) to eliminate the over-inference caused by genetic background differences to the greatest extent.

In the field of lncRNAs, yak research is undergoing a profound transformation from traditional short-read sequencing to the integration of long-read full-length sequencing and single-cell transcriptomics technologies, aiming to achieve comprehensive optimization of genome annotation and high-resolution analysis of regulatory networks. Existing data strongly suggest that lncRNAs deeply penetrate and dominate the core processes of the yak’s low-oxygen physiological adaptation, angiogenesis and tissue remodeling, skeletal muscle evolution, and lipid metabolism reprogramming. Among them, the cis and trans target inference and ceRNA topological network construction remain the most mainstream mechanism explanation framework. Particularly commendable is that the regulatory axis represented by Lnc-MEG8–miR-22-3p–RTL1 has successfully achieved a complete closed loop covering subcellular spatial localization, molecular physical interaction, gene function intervention, and terminal phenotypic detection, setting a normative benchmark for subsequent lncRNA research of “from network inference to causal mechanism”. Despite this, yak lncRNA research still faces systematic deficiencies such as fragmented annotation platforms, extremely low overlap rates of molecular sets across cohorts, and the lack of verification of the biological prerequisites for ceRNA function (including spatial co-localization, absolute expression abundance, and chemical dose-effect). The severe lack of in vivo causal validation evidence makes the transformation path of many candidate molecules into “usable intervention targets or practical molecular markers” extremely difficult.

To promote the fundamental leap of yak ncRNA research from “descriptive association maps” to “conclusive causal mechanisms and industrial application transformation”, future scientific research efforts need to be deeply expanded along the following five core dimensions: First, strive to build a high spatiotemporal resolution and highly cross-platform comparable ncRNA expression panorama, strategically filling the research blind spots of core target organs for plateau adaptation such as the heart, lungs, vascular network, and immune center, and incorporate altitude gradient models, seasonal nutritional fluctuations, and hypoxia and cold stress models into the standardized sampling system. Second, vigorously promote the international standardization of methodological and mechanistic evidence chains. In the functional analysis of the three major types of ncRNAs, it is necessary to forcibly establish a hierarchical empirical norm of “structural confirmation and spatial localization, physical interaction confirmation, causal functional perturbation, rescue experiments, and terminal phenotypic readout” to comprehensively enhance the independent repeatability and cross-data integration capabilities of scientific research results. Third, break the ideological constraints of the single ceRNA theoretical framework. Actively introduce multi-dimensional functional evidence such as RBP complex interactions, endogenous polypeptide translation potential, chromatin epigenetic modifications, and nuclear transcriptional intervention, thereby perfectly resolving the paradox of “inconsistency between expression abundance and phenotypic function” caused by tissue microenvironment heterogeneity and complex feedback loops. Fourth, comprehensively enhance the integration of multi-omics and the precision of single-cell analysis. Deeply integrate single-cell RNA sequencing with high-spatial-resolution transcriptomics technology to precisely identify the specific cell subpopulations where key ncRNA functional axes occur and their exact coordinates in the intercellular communication network. Fifth, take industrial transformation and application as the ultimate goal. Closely focus on the economic and adaptive traits of the plateau, such as IMF deposition efficiency, maintenance of high-quality meat quality, polar reproductive performance, and extreme hypoxia tolerance. Conduct stability and efficacy verification of core candidate ncRNAs in independent validation populations and multiple production scenarios, and steadily create target panels that can directly serve molecular-assisted breeding assessment and refined production management. Through the systematic advancement of the above strategic paths, the field of ncRNA in yaks will surely be refined from the stage of network inference based on massive big data into a theoretical cornerstone for understanding the adaptive evolution of plateau mammals, and provide an indispensable underlying molecular engine for the leapfrog improvement of modern yak breeding.

## 7. Conclusions

In summary, ncRNAs are master regulators governing yak adaptation to extreme environments and the development of key economic traits. This review highlights the pivotal roles of circRNAs, miRNAs, and lncRNAs, demonstrating that post-transcriptional networks converge on critical pathways such as lipid metabolism, nutrient sensing, and hypoxia response. While research has advanced from profiling to mechanism validation, standardization and causal evidence remain bottlenecks. Future efforts should focus on high-resolution spatiotemporal mapping and rigorous functional verification to translate these findings into molecular breeding strategies for plateau animal husbandry.

## Figures and Tables

**Table 1:** A Systematic Summary of Previously Published CircRNA Expression Profiles Across Different Yak Tissues and Experimental Models, Highlighting the Identification Scale, Differential Expression Patterns, and Enriched Functional Themes.

NO	Organization/Model	Grouping/Comparative Design	Total Number of circRNA Identified (N_Total) circRNA	Differential circRNA Number (N_DE, Example Comparison)	Main Enrichment Themes (3–5 Keywords)	Reference
1	Longissimus dorsi (LD)	Different feeding systems (such as grazing vs. supplementary feeding/pen rearing)	12,586	1024	Muscle development; insulin signaling; mTOR Lipid metabolism	[60]
2	Muscle (IMF)	High vs. Low IMF Content	Unreported/unclear (NR)	270 (129 up/141 down)	PPAR-related processes; fatty acid metabolism; adipocyte differentiation; lipolysis; mitochondrial function	[61]
3	primary preadipocytes	Differentiation time series (0, 3, 6, 9 days)	6941	328 (3 days vs. 0 days)	Lipogenesis; Wnt signaling; cell cycle; ECM–receptor interaction; TGF-β pathway	[62]
4	Mammary gland	Lactation period vs. Dry period	11,203	789	Lactation; prolactin signaling; tight junction; milk protein synthesis; immune regulation	[63]
5	Testis	Developmental stages (fetal stage, prepubertal stage, pubertal stage, adulthood)	14,365	2156 (multiple pairwise comparisons)	Spermatogenesis; Meiosis; Hormone response; RNA binding; Chromatin remodeling	[64]
6	Epididymis	Yaks vs. Cattle yaks	1298	294	Sperm maturation; Oxidative phosphorylation; Innate immunity; Cell adhesion; Apoptosis	[65]
7	Muscle + adipose tissue	High vs. Low IMF Deposition	10,477 (combined total of the two organizations)	1362 (tissue-specific)	Lipid storage; myogenesis; PI3K-Akt signaling; extracellular matrix organization	[66]
8	Testis	The age sequence (3, 6, 12, 24 months old)	13,820	1873 (24-month-old vs. 3-month-old)	Reproductive cell development; steroidogenesis; cell proliferation; ubiquitin-mediated protein degradation; spliceosome	[67]

Note: IMF, intramuscular fat. This table summarizes previously published circRNA datasets from independent studies on yaks. N_total and N_DE represent the total number of identified circRNAs and the number of differentially expressed circRNAs, respectively, as reported in the original literature. NR indicates that the result was not reported or was difficult to obtain clearly from the original text. Due to differences in library preparation and bioinformatic pipelines among studies, direct cross-study comparison should be interpreted with caution.

**Table 2:** Yak miRNA–Target Gene Regulatory Axes: Directly Verified Evidence and Phenotypic Associations (Strong Evidence).

Module/Biological Process	miRNA	Direct Target Gene	Model/System	Key Validation Evidence (Core Experiment)	Main Phenotype/Output Indicator	Main Conclusion	Reference
High-altitude adaptation/Hypoxia-induced apoptosis	miR-2285o-3p	CASPASE-3	Comparative study of multiple tissues in yaks and cattle; hypoxia cell model; reporter gene system	Differential expression + miRNA overexpression/inhibition (gain/loss) + apoptosis-related functional experiments + 3′UTR dual-luciferase (WT/Mut) verification of direct targeting	Apoptosis-related indicators (such as apoptosis rate/cell damage indicators, etc.)	miR-2285o-3p directly inhibits CASPASE-3, and attenuates the hypoxia-induced apoptosis/damage phenotype, suggesting its involvement in hypoxia tolerance and high-altitude adaptation-related protective effects	[77]
IMF/adipogenesis	miR-2400	SUMO1	Primary preadipocytes of yaks; reporter gene system	miRNA Mimic/inhibitor intervention + molecular detection (qPCR/WB) + 3′UTR dual-luciferase (WT/Mut) validation of direct targeting + cell proliferation/differentiation function experiments	Proliferation (such as CCK—8/EdU etc.); differentiation (Oil Red O, TG, adipogenic markers)	miR-2400 directly targets SUMO1: promoting preadipocyte proliferation and inhibiting adipocyte differentiation; related to IMF deposition and meat quality.	[78]

Note: This table only includes regulatory axes that simultaneously meet the following conditions: ① There is direct targeting verification of miRNA to the target gene (such as 3′UTR dual-luciferase WT/Mut). ② There is a functional phenotype readout consistent with the related biological process (such as proliferation, differentiation or hypoxia/apoptosis-related indicators). Network association evidence obtained based on transcriptome/enrichment analysis, etc. (omics/predictive evidence), is not included in this table and has been summarized in the “Predicted/omics-based modules” in Figure 2 [74,79,80,81].

**Table 3:** Summary of Representative Studies on Three Major Themes of Yaks lncRNAs (Hypoxia Adaptation, Skeletal Muscle Development, and Lipid Metabolism).

Theme	Reference	Tissue/Model	Experimental Grouping and Control Strategy	Major Methods and Analytical Strategies	Key Findings	Evidence Type
Hypoxia adaptation (muscle/energy metabolism)	[93]	Gluteus muscle (*gluteus*)	Yak, Tibetan cattle, Sanjiang cattle, Holstein cattle; 60-month-old healthy females; 3 individuals per group (n = 3/group) Genomic inference + RT-qPCR validation	Illumina RNA-seq (HiSeq 4000, PE150); lncRNA identification (Cuffcompare + CNCI/CPC); differential analysis (DESeq2, FDR < 0.05 and FC > 2); *cis* (±100 kb) and *trans* (RNAplex, -e < 20 and excluding same chromosome); GO/KEGG; random 10 lncRNAs validated by RT-qPCR	Identified 1364 novel lncRNAs; significant differences in lncRNAs between yaks and other cattle breeds. Trans target gene enrichment suggests energy metabolism and muscle contraction-related processes may participate in high-altitude adaptation, and proposes transcriptional clues for metabolic strategies, fatty acid oxidation and inhibiting glycolysis in yaks	Omics inference + RT-qPCR consistency verification
Hypoxia adaptation (lung tissue)	[100]	Lung tissue	9 male yaks (3 each at 3400/4200/5000 m) + 2 male low-altitude control cattle (1500 m, n = 2)	mRNA/lncRNA RNA-seq + miRNA sequencing; differential analysis (edgeR, multiple comparisons, BH correction); *cis* (<50 kb) + *trans* (correlation); GO/KEGG; ceRNA network construction; RT-qPCR validation (7 mRNA + 5 lncRNA)	Systematically revealed differential expression profiles between yak and low-altitude cattle, and among yaks at different altitudes; enrichment and network analysis suggest fatty acid metabolism, immune response, and cell cycle processes participate in lung tissue hypoxia adaptation	Omics inference + ceRNA network + RT-qPCR consistency verification
Hypoxia adaptation (cardiovascular cell model)	[101]	Yak coronary artery smooth muscle cells (CASMCs)	Normoxia 21% O_2_ vs. hypoxia 5% O_2_; transcriptome libraries: n = 3 for normoxia, n = 3 for hypoxia; CCK-8 n = 4; PCNA Western blot n = 3	Cell identification (α-SMA, calponin-1 positive; CD31 negative; >98% smooth muscle cells); CCK-8 and PCNA validation of proliferation; whole transcriptome sequencing (Illumina HiSeq 4000; mRNA/lncRNA + small RNA libraries); differential analysis (mRNA/lncRNA: DESeq2; miRNA: edgeR; FC ≥ 1.5 and *p* < 0.05); GO/KEGG; ceRNA network (TargetScan/miRanda + hypergeometric test and Pearson correlation); Cytoscape; qRT-PCR validation (11 mRNA, 11 lncRNA, 8 miRNA)	Hypoxia promotes CASMC proliferation; identified 835 differential mRNAs, 285 lncRNAs, and 126 miRNAs; enrichment points to ECM, immunity, metabolism, cell development processes and TGF-β, MAPK, cAMP, mTOR, PI3K-Akt pathways; constructed ceRNA network and screened core regulatory elements related to hypoxia adaptation, with qRT-PCR trends consistent with sequencing	Cell phenotype + omics inference + ceRNA network + qRT-PCR consistency verification
Muscle development/IMF deposition	[90]	Longissimus dorsi (LD)	18 male Tianzhu white yaks: n = 6 each at 6/30/54 months	RNA-seq (NovaSeq); lncRNA identification (CPC2/CNCI); differential analysis (DESeq2, *p* < 0.05 and |log_2_FC| > 1); STEM trend analysis; *cis* (±100 kb) + *trans* (correlation); GO/KEGG; co-expression network; random 11 lncRNAs validated by RT-qPCR	log2FC	>1) STEM trend; *cis* (±10 kb) + *trans* (correlation); GO/KEGG; co-expression network; random 11 lncRNAs RT-qPCR validation
Muscle development (fetal–juvenile–adult)	[97]	Longissimus dorsi	9 female polled yaks: n = 3 each at fetal 90 days/6 months/3 years	RNA-seq (HiSeq 2500); lncRNA identification (multi-tool filtering); differential lncRNAs (padj < 0.001 and |log_2_FC| > 1.2); *cis* (±100 kb) + *trans*	log2FC	>1.2); *cis* (±100 kb)+ *trans*
Muscle resource/annotation enhancement (yaks vs. cattle–yaks)	[103]	Longissimus dorsi (LD)	Yak Y1–Y3 vs. cattle–yak CY1–CY3; all 4 years old, same environment; 3 individuals per group (n = 3/group)	ONT PromethION full-length transcriptome; novel transcript annotation (Nr/SwissProt/GO/KEGG/KOG); AS identification (SUPPA); lncRNA prediction (CPC + CNCI + SwissProt intersection); differential transcripts (DESeq2, FDR < 0.05 and |log_2_FC| > 1)	log2FC	>1) PPI (STRING/Cytoscape); PCR/Sanger and RT-qPCR validation
Muscle development mechanism validation (ceRNA axis)	[91]	Yak primary myoblasts (from LD) + multi-tissue expression profile Functional validation (phenotype + molecular mechanism)	Cells: MEG8-OE/OE-NC, Sh-MEG8/Sh-NC; miR-22-3p mimic/inhibitor; RTL1 OE/knockdown and control; multiple annotations: 3 biological replicates (n = 3); tissues: multiple tissues from 3-year-old female yaks (n = 3)	Percoll gradient separation and purification; nuclear/cytoplasmic separation qRT-PCR and RNA-FISH localization; PI cell cycle, EdU, CCK-8 proliferation; *MYF5*/*MYOG* qRT-PCR and WB; immunofluorescence myotube formation; dual luciferase validation of targeting relationship and rescue experiments	*MYF5*/*MYOG* and Myotube Formation	Functional verification (phenotype + molecular mechanism)
Lipid metabolism (liver/age stages)	[92]	Liver	9 Maiwa yaks; LD: 1-day-old n = 3, LM: 15-month-old n = 3, LY: 5-year-old n = 3; consistent feeding management; liver collected immediately after slaughter and stored in liquid nitrogen	Strand-specific RNA-seq (rRNA removal; Illumina HiSeq, PE150); alignment (HISAT2 v2.0.5); assembly and quantification (StringTie; FPKM); differential analysis (DESeq2, padj < 0.05); lncRNA target gene prediction (co-location + co-expression); co-expression network and functional enrichment (clusterProfiler GO/KEGG); qRT-PCR validation (6 lncRNAs + 7 mRNAs, β-actin as internal reference; further validation of TCONS_00098792 and target genes)	mRNA 35,216 and lncRNA 10,073 were detected; 288 differentially expressed lncRNAs were identified and 88 lncRNAs related to metabolism were screened out, which were inferred to be involved in lipid metabolism, collagen remodeling and protein transport. The trend of qRT-PCR and sequencing differences was highly consistent, supporting the application of the established “transcriptome + co-expression network” process for the screening of functional lncRNAs in non-model species.	Omics inference + co-expression network + qRT-PCR validation
Fat deposition (feeding system differences; whole transcriptome + ceRNA)	[104]	Subcutaneous fat (12th–13th rib level)	6 healthy male Huanhu yaks (2 years old, 210.33 ± 10.23 kg); GF n = 3 vs. SF n = 3; trial period 6 months; fasting 24 h and water deprivation 8 h before slaughter Genomic inference + ceRNA network + qPCR validation	Whole transcriptome: mRNA/lncRNA/circRNA (rRNA-depleted strand-specific library, HiSeq 2500, PE150) + small RNA library (HiSeq 2500); alignment and assembly (HISAT2 + StringTie); lncRNA (cuffcompare “i/u/x/o” + >200 bp and exons > 2 + CPC/CNCI/PLEK/Pfam); circRNA (BWA + CIRI); miRNA (miRBase21 + miRDeep2); differential (DESeq2, FC > 2 or <0.5 and q < 0.05); GO/KEGG; ceRNA network (Pearson ≥ 0.80 and *p* < 0.05 + hypergeometric test); qPCR validation (7 RNAs)	Enrichment and network analysis indicated that pathways such as PPAR, PI3K-Akt, and cAMP were involved in the regulation of fat deposition; TCONS00042948, TCONS00012083/bta-miR-2316/MCAT and NR4A3 were proposed. enrichment and network analysis suggest PPAR, PI3K-Akt, cAMP pathways participate in fat deposition regulation; proposed key candidate axes including TCONS_00042948, TCONS_0012083/bta-miR-2316/MCAT and NR4A3, with qPCR trends consistent with sequencing	Omics inference + ceRNA network + qPCR consistency verification
IMF/Hypoxia crossover (HIF1α functional validation)	[102]	Longissimus dorsi (LD) + yak intramuscular preadipocytes	Tissues: 24 Xiaojin county yaks measured for IMF, 6 selected for sequencing; H-IMF n = 3 (4.50 ± 0.20) vs. L-IMF n = 3 (2.52 ± 0.11); cell experiments: three biological replicates, three technical replicates	RNA-seq (mRNA/lncRNA, HiSeq 4000) + miRNA sequencing; differential screening: lncRNA/mRNA (DESeq2) and miRNA (edgeR), threshold FC > 1.5 and *p* ≤ 0.05; lncRNA target gene prediction (antisense: RNAplex; *cis*: ±10 kb; *trans*: co-expression + LncTar); miRNA target genes (miRanda + TargetScan intersection); ceRNA network (miRNA–ceRNA Spearman ≤ −0.7; ceRNA pair Pearson > 0.9; hypergeometric test *p* ≤ 0.05; Cytoscape); qRT-PCR validation (5 mRNA, 4 lncRNA, 1 miRNA); HIF1α function: adenovirus overexpression (Ad-HIF1α) and siRNA interference (si-HIF1α) + CoCl_2_ simulated hypoxia (200 μM); CCK-8 proliferation, Oil Red O staining and TG determination, qRT-PCR of adipogenic marker genes	To construct the differential spectra of mRNA/miRNA/lncRNA and ceRNA network of LD in yaks at different IMF levels and screen the candidate regulatory axes; meanwhile, through the overexpression/interference of HIF1α combined with proliferation and lipid droplet formation detection, it is indicated that HIF1α participates in regulating the proliferation of intramuscular preadipocytes and lipid droplet formation in yaks, providing mechanism evidence for the cross-regulation of “hypoxia signal–IMF deposition”.	Omics inference + ceRNA network + functional validation (cellular)

Notes: GF = grazing feeding, SF = stall feeding, IMF = IMF, LD = longissimus dorsi, CASMCs = coronary artery smooth muscle cells, DE = differentially expressed, *cis*/*trans* = *cis*/*trans* target gene prediction, ceRNA = competitive endogenous RNA network; n represents the number of biological replicates (unless otherwise specified in the text).

## Data Availability

Data availability is not applicable to this article as no new data were created or analyzed in this study.
